# The maps of meaning consciousness theory

**DOI:** 10.3389/fpsyg.2024.1161132

**Published:** 2024-03-22

**Authors:** Scott Andersen

**Affiliations:** ^1^United States Department of Homeland Security, Washington, DC, United States; ^2^Liberty University, Lynchburg, VA, United States

**Keywords:** consciousness, animal model, human model, prospection, personality, intelligence quotient, perception, inhibition

## Abstract

In simple terms, consciousness is constituted by multiple goals for action and the continuous adjudication of such goals to implement action, which is referred to as the maps of meaning (MoM) consciousness theory. The MoM theory triangulates through three parallel corollaries: action (behavior), mechanism (morphology/pathophysiology), and goals (teleology). (1) An organism’s consciousness contains fluid, nested goals. These goals are not intentionality, but intersectionality, via the Darwinian byproduct of embodiment meeting the world, i.e., Darwinian inclusive fitness or randomization and then survival of the fittest. (2) These goals are formed via a gradual descent under inclusive fitness and are the abstraction of a “match” between the evolutionary environment and the organism. (3) Human consciousness implements the *brain efficiency hypothesis,* genetics, epigenetics, and experience-crystallized efficiencies, not necessitating best or objective but fitness, i.e., perceived efficiency based on one’s adaptive environment. These efficiencies are objectively arbitrary but determine the operation and level of one’s consciousness, termed as *extreme thrownness*. (4) Since inclusive fitness drives efficiencies in the physiologic mechanism, morphology, and behavior (action) and originates one’s goals, embodiment is necessarily entangled to human consciousness as it is at the intersection of mechanism or action (both necessitating embodiment) occurring in the world that determines fitness. (5) Perception is the operant process of consciousness and is the *de facto* goal adjudication process of consciousness. Goal operationalization is fundamentally efficiency-based via one’s unique neuronal mapping as a byproduct of genetics, epigenetics, and experience. (6) Perception involves information intake and information discrimination, equally underpinned by efficiencies of inclusive fitness via extreme thrownness. Perception is not a ‘frame rate’ but Bayesian priors of efficiency based on one’s *extreme thrownness*. (7) Consciousness and human consciousness are *modular* (i.e., a scalar level of richness, which builds up like building blocks) and *dimensionalized* (i.e., cognitive abilities become possibilities as the emergent phenomena at various modularities such as the stratified factors in factor analysis). (8) The meta dimensions of human consciousness seemingly include intelligence quotient, personality (five-factor model), richness of perception intake, and richness of perception discrimination, among other potentialities. (9) Future consciousness research should utilize factor analysis to parse modularities and dimensions of human consciousness and animal models.

## Introduction

Science progresses through both theory and experiment. Moreover, theory precedes experiment *a priori* and guides empirical research (Seth and Bayne, 2022). This article seeks to propose a theory of consciousness for empirical research based on a first-principles conceptualization of the notion of consciousness. This theory seeks to address these notions that are insufficiently covered in many current conceptualizations of consciousness:

- Evolutionary biology acted as the mechanics for the development of consciousness, i.e., random genetic variation, the organism’s embodiment meeting the world to subsequently determine fitness and then survival of the fittest in a constantly changing environment.
- *Occam’s Razor*: Just because the effects of something may be profound, neither the explanation nor the mechanics behind such effects need to be profound (Stanford Encyclopedia of Philosophy, 2022a). To my knowledge, this notion is commonly lacking in the discussions of consciousness, as consciousness is assumed by some to be a magical phenomenon and/or unique to the complexity of the human mind. I seek to enlighten the use of these notions.
- *Hume’s Dilemma*: One cannot derive an *ought* from an *is* (Stanford Encyclopedia of Philosophy, 2022b). It seems that most of the discourse on consciousness imposes an *ought* on human consciousness, as some unique spark of the divine exclusive to humanity, This perception perhaps could simply be attributed to Kahneman’s (automatic) System 1 thinking (Kahneman, 2011) or McGilchrist predominance of left-brain thinking societally, generally in the current times (McGilchrist, 2019), and I seek to clarify this notion.

Equally, it is important to keep in mind the appropriate level of empiricism associated with the scientific, well-defined notion of a *theory*. A theory is broadly conceptualized as a construct or system of ideas that collectively seek to explain a phenomenon in the world, independent of the other phenomena. Essentially, one could think of a theory as a unique, emergent phenomenon from a certain series of ideas (Robson and McCartan, 2016). For example, Darwin’s theory of natural selection consisted of the ideas of random genetic variations and how the matching of those random variations to an environment leads to *fitness*, whereby such a fitness leads to increased offspring production compared to those random genetic variations lacking that fitness, which could be referred to as the *survival of the fittest*. Overall, currently, random genetic variation, with a subsequent fitness to the environment and then ultimately leading to the survival of the fittest, is the theory of *natural selection*.

## Consciousness defined

In simple terms, consciousness should be seen as the irrevocable (and unexceptional) byproduct of having multiple goals for action in the world and the process by which one continuously adjudicates across such goals to implement action continuously in the world.

## Key concepts

### Fluid hierarchy of goals

• The fundamental constitution of consciousness involves having multiple goals and continuously adjudicating across such goals so as to operationalize action continuously in the world.
o One can think of an organism’s consciousness as containing a fluid, but a nested, hierarchy of goals.
o These goals are not a matter of intentionality, but intersectionality, via the Darwinian byproduct of embodiment meeting the world. It is a matter of inclusive fitness, i.e., random variation and then survival of the fittest.
▪ For all intents and purposes, the MoM consciousness theory teleologically conceptualizes these goals as representations of a “match” of inclusive fitness to an adaptive evolutionary environment, past or present, though apomorphy, which may equally originate from the genetic fringes.

• These goals have a corollary action in the world and a corollary mechanism among the organism (e.g., the need for energy for a human has the corollary action of obtaining food in the world and is activated mechanistically and biochemically via the hormone Ghrelin, among other mechanisms and complexities).

• Some goals have simple corollary actions and mechanisms, while other goals have complex corollary actions and mechanisms. The continuous process of adjudicating one’s nested hierarchy of goals makes it possible for the organism to manifest any of its goals, at any time, along with the subsequent action as mediated mechanistically under the right conditions.
o Some corollaries of goals, mechanisms, and/or actions are fractals among the organism, i.e., the same phenomena, but manifesting differently at multiple levels of analysis.
▪ This is a phenomenon demonstrated beyond human models and beyond reproach by the work of Dr. Michael Levin and his lab (Tufts University, 2023) and Dr. Josh Bongard and his lab (Kudithipudi et al., 2022), referred to as *multilevel competencies*.
o These multilevel competencies (and subsequent goals) begin at the most fundamental levels of biology (Levin, 2023a, 2023b).
▪ The most fundamental of the organism’s goals of addressing entropy subsequently appears for humans, fractally at the individual neuron and the whole of the brain, which fundamentally crystallizes prediction-to-outcome matches in the world (i.e., prospection) so as to reduce entropy and free energy of thinking and behaviors of the organisms in the future (Carhart-Harris et al., 2014).
▪ Moreover, from the very start of the earthly phylogenetic tree, the need for energy manifests intrinsically among organisms, nested upon the presuppositions of both homeostasis and replication, with the corollary action of movement primitively (Kumar and Philominathan, 2010; Swiecicki et al., 2013). Then, the complex physiological mechanism later manifests as, for example, hunger in humans.
▪ Numerous examples of this phenomenon are observed; for instance, in human pathophysiology, the renin–angiotension system is a higher order representation of mechanistic osmotic equilibration at the cellular level.

• The organism’s goals, hierarchy of goals, and means by which to adjudicate across such goals continuously (goal adjudication conceptualized in this theory as “perception”) are the modest byproducts of the experiential existence in which an organism or an organism’s consciousness finds itself.
o Heidegger referred to the randomness of the time and place in which your consciousness finds itself as *thrownness* (Heidegger, 2008).
o Equally, one should conceptualize that the very specific time and place in which consciousness finds itself also determines how that very consciousness develops and the meanings it endows on the world as such, i.e., genetics, epigenetics, and experience further shape one’s goals, Bayesian priors, neuronal architecture, and subsequently consciousness. The MoM consciousness theory terms this concept *extreme thrownness.*

### Consciousness entangles embodiment

• One can think of an organism’s consciousness as containing a fluid, but nested, hierarchy of goals.
o These goals are not a matter of intentionality, but intersectionality, via the Darwinian byproduct of embodiment meeting the world. It is a matter of inclusive fitness, i.e., random variation and then survival of the fittest.
▪ For all intents and purposes, the MoM consciousness theory teleologically conceptualizes these goals as representations of a ‘match’ of inclusive fitness to an adaptive evolutionary environment, past or present.

• Consciousness at its core is the mapping of our embodiment onto the world, which forms the very goals and hierarchy of the goals of consciousness because it is the environment that determines which Darwinian random variations survive to become the fittest.
o One might argue if embodiment is *ipso facto* required for consciousness, and validly so, but it is beyond reproach to conceptualize earthly and subsequently human consciousness as anything other than what is necessarily entangled to embodiment.

• This theory speaks purely to the notion of earthly, evolutionarily derived consciousness, as instantiated particularly in human experience or Heidegger’s concept of *dasein* (Heidegger, 2008), often referred to in the research body as *phenomenological consciousness* (Carruthers, 2019, p. 41).
o The entanglement of consciousness and embodiment as such makes it beyond the scope of this theory to apply this conceptualization of consciousness to artificial intelligence. While, evidently, the MoM consciousness theory should apply to artificial intelligence, the latter is not subject to the same notion of embodiment or its direct mapping onto the world with the very mechanism of goal orientation occurring via natural selection.
▪ One could equally (perhaps necessarily) argue for an empirical study of the consciousness of artificial intelligence utilizing the MoM consciousness theory.
o Additionally, if one can conceptualize *gradual descent* or *gradient descent* (i.e., continuous complexification from optimization to a changing environment) as integral to and the very underpinnings of the evolutionary-derived and evolutionary-directed process of inclusive fitness, a brain or likely even a nervous system, which, ergo, is not required for complex behaviors to be associated with consciousness (Ryan and Grant, 2009).
▪ For instance, Botton-Amiot et al. (2023) demonstrate that the anemone sea starlet species *N. vectensis* is able to form associative memory when subjected to classical conditioning, i.e., this is a simple sea organism absent of the central nervous system, demonstrating learned behavior and memory. Botton-Amiot et al. (2023) state “these results root associative learning before the emergence of [*nervous system*] centralization in the metazoan lineage and raise fundamental questions about the origin and evolution of cognition in brainless animals” (p. 1).
▪ Levin (2023a, 2023b) equally and empirically demonstrates goals and the Bayesian priors of associative learning from these goals as much more fundamentally stored among every organism than purely among the centralized nervous system.

### Perception is goal adjudication

• The fundamental constitution of consciousness involves having multiple goals and continuously adjudicating across such goals so as to operationalize action continuously in the world.
o Perception is *de facto* the operant process of consciousness and is the very process of goal adjudication.

• The process of goal adjudication through consciousness (i.e., perception) involves components of both information intake and information inhibition/discrimination (Carruthers, 2019).
o The intake of information for perception involves not just raw information intake (e.g., vision) but also systems of value judgments that are patterns based on genetics and epigenetics (e.g., IQ and personality) and experience (e.g., learned patterns and behaviors) of the organism or organism’s consciousness that equally narrows the process of raw information intake to the simplest operable conceptualizations of understanding.
▪ For example, an individual’s understanding of a helicopter is sufficient for how they personally act in the world, though they likely could not fly, nor fix, nor explain in detail the mechanics of how a helicopter works.

• *Perception* should likely be conceptualized as a key factor or a *dimension* of consciousness, which is comprised of multiple sub-dimensions of consciousness as organisms maintain multiple constructs of sensory perception (Birch et al., 2020), as the very goal-oriented decisions that organisms make (to include humans) and is evolutionarily derived and evolutionarily directed toward an implicit notion of value judgment or goal rank-ordering in perception. See Birch et al. (2020) for an example of a dimensionalized construct of animal consciousness.
o For example, why do you not stare at one single molecule of one single object, endlessly for the entirety of one’s life, as that singular thing contains an infinite amount of complexity that could never exhaust one’s perception during one’s lifespan?

• The process of the brain seeking to be as efficient as possible in information discrimination is known as the *brain efficiency hypothesis*, a well-established finding in neuroscience (Basu et al., 2022).
o These very attempts at efficiency are a byproduct of *extreme thrownness*, i.e., these efficiencies from the organism’s goals, hierarchy of goals, and means by which to adjudicate across such goals continuously (known as perception) are a product of the environment in which an organism or an organism’s consciousness finds itself.

### Evolutionary derived and evolutionarily directed

• The fundamental constitution of consciousness involves having multiple goals and continuously adjudicating across such goals so as to operationalize action continuously in the world.
o These goals are the modest byproducts over time of the optimization strategies that organisms utilize in various environments, with a tendency for increasing complexity over time due to continuous optimization to a changing environment. The trend of increasing complexity over time from continuous optimization is a phenomenon known as *gradient descent* or *gradual descent* (Theodoridis, 2015).
o This operant and differing goal-seeking and adjudication of consciousness, being most fundamentally evolutionarily derived and evolutionarily directed, scales from the most basic goal of matter in combating entropy (Shcherbakov, 2005) and the most basic goals of survival at the cellular and sub-cellular levels.
▪ Operationalizing the competing goal of survival quintessentially and cellularly further stratifies into the survival mechanisms of homeostasis (during states of low hospitability) and replication (during states of high hospitability), which stand opposite to the perspectives of action in the world (Sinclair and LaPlante, 2019).
o As organisms scale to various higher levels of complexity, the organism’s goals also scale to more nested, more complex differing goals, creating highly complex, highly nested rank-orders of differing goals in the world for the organism (though all fundamentally nested toward addressing entropy, further stratified into the tensioned, cellular goals of homeostasis and replication).

• These goals form not as a matter of intentionality, but intersectionality, via the Darwinian by-product of embodiment meeting the world via inclusive fitness, i.e., random variation and then survival of the fittest.
o While some goals in the hierarchy are more readily operationalizable or useful, human consciousness is thoroughly filled with “ghost in the machine” goals, which may become operationalized when the appropriate threshold of mechanistic conditions arise
▪ exemplified by the mismatch of human taste preferences to the modern environment (Breslin, 2013),
▪ exemplified by the duality of the human mind, which seeks efficiency preeminently (Basu et al., 2022), manifested through the mode of thinking of simplification of the world (Kahneman, 2011; McGilchrist, 2019), and replete with cognitive heuristics, biases, and fallacies.
▪ empirically exemplified by Schaffner et al. (2023) who demonstrate that sensory perception and its Bayesian encoding of priors so as to tune perception as a matter of fitness maximization.

### Modular and dimensionalized

• Human consciousness is centered around the central nervous system (i.e., the brain), and the functional unit of the brain is the neuron. The neuron’s goal, corollary to the mechanism, is the prediction of outcomes followed by the process of plasticization based on the predication-to-outcome match or mismatch in the world (Wacongne et al., 2011; Pitts et al., 2018; Carruthers, 2019; Pereira et al., 2021). This concept is commonly referred to in the literature as *prospection* (Carruthers, 2019).
o The process of prospection and subsequent plasticization in one’s brain builds networks (mechanistically) and patterns of behavior (the corollary action) for operationalization based on how one’s brain develops. These networks and patterns may not necessarily be what is best, right, objective, or most useful. We operate an internally and experientially built model of the world, we operate *maps of meaning*.
o Pioneering neuroscientist Sokolov (2001) wrote decades ago about this, with one’s orienting reflex acting as a kind of adjudication mechanism as such between our internal model(s) of the world and the actuality of the world around us.
o Additionally, the recent work of Cazettes et al. (2023) in the neuroscientific study of mice found that whatever behavior a mouse implemented and subsequently, regardless of the explanatory process the mice utilized to implement the said behavior, the secondary motor cortex of such mice still encoded the full set of possible behaviors for the situation, i.e., the mice essentially simulated all the behaviors, encoded such into memory, and then acted in the manner they best saw fit.

• As various complexities of goals and goal hierarchies form, along with subsequently more and more complex processes of perception, the human brain equally develops more and more complexity of neuronal networks (the mechanism) and cognitive abilities (the corollary action).
o Braddick (2001) and Sapolsky (2005) annotate these neuronal networks, as they go from inner layers to outer layers and encode more specific information, which could equally be described as attributed to more complex goal schemas and Bayesian priors.

• As certain levels of complexity of consciousness and subsequently the human brain form, termed *modularities* in the MoM consciousness theory, certain cognitive abilities simply emerge as the emergent phenomena (Gruber and Voneche, 1977; Carruthers, 2019; Birch et al., 2020).
o For example, Piaget discovered that the cognitive ability of *conservation* emerges (the action) around in children when their brains have reached the corollary level of complexity (mechanistically via prospection) (Gruber and Voneche, 1977).
▪ Even if Piaget’s conservation is learned, a finding not exactly known or most likely currently, it still stands that a pre-requisite level of cognitive complexity must be met so as to “learn” conservation, i.e., emergence is not pre-determined, but as a set of possibilities at the necessary pre-requisite complexity. This is the *neural pre-requisites* line of thinking within the research body.

• These emergent phenomena that simply emerge at varying modularities of consciousness are termed *dimensions* in the MoM consciousness theory.
o Dimensions of human consciousness have sub-dimensions, and these sub-dimensions have further sub-dimensions, and so on.
o One may think of consciousness and human consciousness as building blocks. Human consciousness has simply built up to a high level of complexity or *modularity* of consciousness, with a vast number of *dimensions* (or corollary goals via cognitive abilities) as a result of the complexification of goals and corollary mechanisms via evolution with a gradual descent.

• Each instantiation of a species’ consciousness must be recognized as a rough type and *sui generis* (Carruthers, 2019) due to the Darwinian landscape in which said consciousness developed. However, since there are genetic, epigenetic, and experiential components to the development of consciousness (via prospection mechanistically), it stands that each organism across a species (e.g., each individual human) additionally may vary in their modularity (and subsequent dimensions) of consciousness.
o Each unique instantiation of consciousness across a species may have been subtly nuanced, and different modularities and/or dimensions of consciousness are *causa sui* of their differing goals, their particular embodiment (and as mapped onto the world), and their unique neural plasticity, all of which have a context of *extreme thrownness.*
▪ Alexander Luria demonstrated how human individuals in rural societies lacked certain abstraction abilities possessed by human individuals in industrialized societies, i.e., their dimensions of consciousness (and modularity) may have been lower due to the extreme thrownness of the time, place, and experiences of how their consciousness developed. He notably discovered this finding through the use of IQ tests, particularly the sub-test of Raven’s Progressive Matrices (Epstein, 2018).

• Conceptually, factors or *dimensions* of consciousness within the research body have been suggested to include, but are not limited to, memory (LeDoux and Lau, 2020), self-awareness, or selfhood (Brown et al., 2019), or attention (Pitts et al., 2018), among other potential factors.

• The MoM consciousness theory instead posits the most metacognitive dimensions of human consciousness likely as richness of perception (intake), richness of perception inhibition or discrimination, and some existing measures in psychology, with perhaps others yet to be determined.
o Intelligence quotient (IQ) is the single most reliable and valid measure of individual differences and human life outcomes (Roberts et al., 2007; Ritchie, 2016; Jarrett, 2021), with IQ being a singular measure of a construct of human cognitive abstraction abilities (referred to as “*g*”).
▪ IQ is a well-established, stratified construct where lower levels and series of cognitive abilities presupposes higher levels and higher series of cognitive abilities, as exemplified by the aforementioned *three stratum theory of cognitive abilities* from Carroll (2005).
▪ Not only does the construct of IQ evince the modularity and dimensionalization of consciousness of the MoM theory, its predictive validity and Alexander Luria’s work suggest the construct as a key component of the human instantiation of consciousness. One could think of IQ as *the measure of richness of one’s prospection abilities*.
o Psychometric measures of personality, particularly as measured via the five-factor model of personality or colloquially *the big five*, is another well-established, stratified construct of individual differences in psychology with high reliability and validity.
▪ The stratification of *the big five* is exemplified by the Big Five Aspect Scale (DeYoung et al., 2007).
▪ Personality is essentially the measure of the metacognitive mosaics of human value judgments and as such seems likely a dimension of human consciousness. One might consider personality as *the measure of the extreme thrownness of one’s consciousness*.

## Future research

• The seemingly right approach to studying consciousness is factor analysis. Factor analysis is a statistical analysis methodology, common in research, that conceptually identifies the unique, big picture concepts among data sets.
o For example, factor analysis is the means by which various notions such as intelligence quotient and measures of personality psychology have been derived among various constructs of various fields.
o Factors, essentially termed *dimensions* in the MoM theory, of human consciousness may include but are not limited to IQ, personality (specifically the five-factor model of personality), perceptual richness, and perceptual inhibition/discrimination richness.

• Ample opportunities are available to study both animal models and human models of consciousness factor analytically.
o For instance, the organism *C. elegans* with a fully mapped nervous system of 302 neurons and extensive research of its epigenetic mechanisms provides unique opportunity to parse a specific modularity (and subsequently dimensions) of consciousness along with the corollary goals, actions, and mechanisms.
▪ *C. elegans* is uniquely suited for consciousness research as presupposed upon the MoM consciousness theory due to the work of Oded Rechavi and others in detailing the epigenetics of *C. elegans.*

## Author contributions

The author confirms being the sole contributor of this work and has approved it for publication.
